# A Rare Case of Incidental Catamenial Pneumothorax With Endometriosis-Related Ascites and Pelvic Endometriosis

**DOI:** 10.7759/cureus.63117

**Published:** 2024-06-25

**Authors:** Feras Al-Moussally, Sesha Kanakamedala, Omar M Masarweh, Saud Khan, Roger Crouse

**Affiliations:** 1 Department of Internal Medicine, University of Central Florida, Orlando, USA

**Keywords:** endometriosis surgery, pneumothorax (ptx), endometriosis diagnosis, endometriosis related ascites, catamenial pneumothroax

## Abstract

Endometriosis is characterized by the ectopic implantation of a functional uterine lining outside of the uterus. Commonly, it occurs in the fallopian tubes, ovaries, uterosacral ligaments, and gastrointestinal tract. Less commonly, it may occur in the pericardium, pleura, and central nervous system. Thoracic endometriosis syndrome includes multiple presentations, most commonly catamenial pneumothorax. We present a case of a catamenial pneumothorax that was incidentally found on imaging after the patient presented with complaints of abdominal pain and weight loss.

## Introduction

Endometriosis is a common condition affecting up to 10% of females worldwide. The manifestations of the disease outside of the reproductive system remain poorly understood [1]. One of these manifestations is thoracic endometriosis syndrome, which includes catamenial pneumothorax, catamenial hemothorax, catamenial hemoptysis, catamenial hemopneumothorax, and endometriosis lung nodules. Catamenial pneumothorax is the most common form of thoracic endometriosis syndrome. It usually occurs in women of reproductive age and is defined as spontaneous recurrent pneumothorax with a temporal relationship to menses [2]. We intend to present a case where a catamenial pneumothorax was incidentally found on imaging.

## Case presentation

The patient was a 38-year-old female gravida 0, para 0, with a previous medical history of endometriosis. She presented to the emergency department with complaints of abdominal pain, nausea, and vomiting for about three weeks with intolerance to both solids and liquids. She had reported that her symptoms had been getting progressively worse with a weight loss of 15 pounds. Her last menstrual period was 25 days prior to the presentation. A review of the systems was otherwise negative, with no shortness of breath.

Her past medical history was significant for an isolated spontaneous pneumothorax four years ago. In addition, she had an extensive history of bowel obstructions, for which she underwent small and large bowel resections with re-anastomosis. At that time, a diagnosis of endometriosis was made two years ago. She was started on leuprolide but was lost to follow-up due to the COVID-19 pandemic.

She denied any other prior medical history, including smoking, drinking, or using any drugs.

In the emergency department, the heart rate was 66 beats per minute, the blood pressure was 124/89 mmHg, the temperature was 36.8 °C, and the respiratory rate was 18 breaths per minute. Her oxygen saturation was 99% in room air. Laboratory testing showed only mild leukocytosis with a WBC of 11 (reference range 4.0 - 10.5 10^3/u); otherwise, they were nonrevealing.

**Figure 1:**
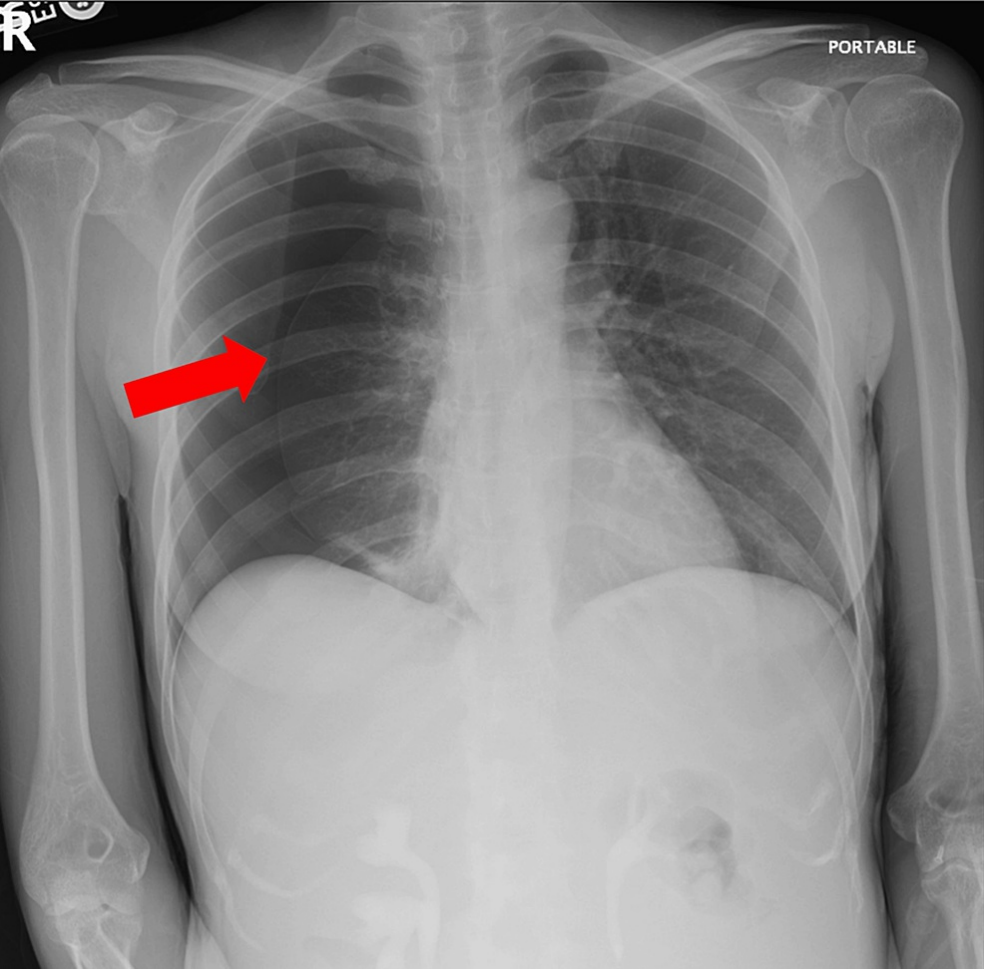
Chest X-ray showing collapsed right lung with arrow indicating the visible pleural edge line

**Figure 2:**
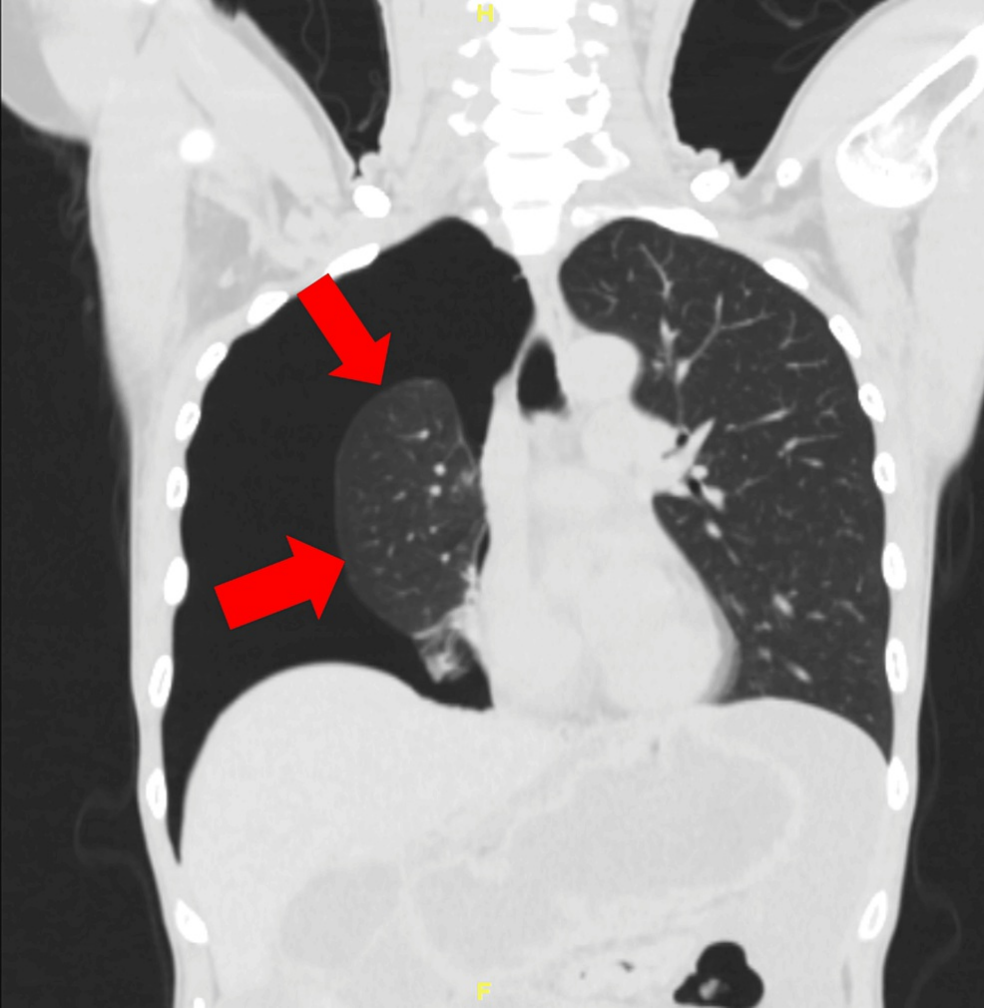
CT chest showing collapsed right lung with arrows indicating the visible pleural edge line

A computed tomography (CT) scan of the abdomen and pelvis with contrast showed a collapsed right lung with a right pneumothorax, extensive heterogeneous pelvic masses and peritoneal lesions, and a large amount of fluid in the abdomen and pelvis. Chest X-ray and CT chest were ordered and showed a large right-sided tension pneumothorax with right lower lobe atelectasis (Figures 1, 2). A right chest tube pigtail catheter was placed, and a repeated chest X-ray showed complete resolution of the right pneumothorax.

She was diagnosed with a partial small-bowel obstruction that resolved spontaneously with conservative management. Paracentesis was done with 1.2 liters of dark red fluid removed. Analysis was suggestive of transudative fluid, and cytological analysis was negative for malignant cells but had blood and macrophages. Laboratory testing revealed a CA-125 level of 470 nanograms per milliliter and a normal carcinoembryonic antigen (CEA).

Due to the history of recurrent pneumothorax, right video-assisted thoracoscopic surgery, apical wedge, total pleurectomy, and chemical pleurodesis were performed for her catamenial pneumothorax. The patient tolerated the procedures well and had a chest tube placed for pneumothorax. Chest tube was initially placed to suction with slow improvement in pneumothorax. Eventually, the chest tube was removed with a stable chest X-ray, and the patient continued to tolerate breathing well on room air. She was discharged home afterward, with plans to follow up with an outpatient.

## Discussion

Endometriosis is a cyclic disease of female endometrial tissue that is growing and functioning outside its usual confines on the inner lining of the uterus. Ectopic endometrial cells maintain their normal reactivity to hormonal cycles of progesterone and estradiol signaling, resulting in a fluctuation of symptoms commensurate with the menstrual cycle. A catamenial pneumothorax is defined as having at least two spontaneous pneumothoraces occurring in close proximity to menstruation. One of the first unequivocal examples of recurrent pneumothoraces related to menses was documented in 1958, when a woman was reported as having recurrent monthly pneumothoraces at the time of menstruation for 12 months in a row. Thoracotomy revealed thoracic endometriosis [3]. Interestingly, the occurrence of catamenial pneumothorax occurs in the right lung 90% of the time. This observation may be explained by the normal clockwise flow of peritoneal fluid in the peritoneal cavity, which carries ectopic endometrial cells from the pelvis up to the hepatic area [4]. Congenital or acquired defects in the diaphragm have also been proposed as mechanisms for ascites migrating into the right chest (hepatic hydrothorax). This could also explain how endometrial cells from the abdominal cavity would preferentially wind up in the right thoracic cavity [5]. The various chest symptoms that occur with thoracic endometriosis could then be explained by where endometrial implants attach themselves, forming chocolate-like cysts. Endometrial tissue that attaches to the visceral pleura could explain a pneumothorax and/or a hemothorax; attachment to the parietal pleura could explain chest pain; attachment to the diaphragm could explain right upper quadrant abdominal pain or pain referred to the right shoulder; and attachment to the tracheobronchial tree could explain hemoptysis. On the other hand, catamenial pneumothorax may occur without symptoms of pain and without findings of abdominal endometriosis [6]. These varied symptoms would be expected to occur at or three days on either side of the start of a menstrual period.

Despite its frequency and present understanding of its pathophysiology, the triggers and causes of catamenial pneumothorax in particular and endometriosis, in general, are still poorly understood. It has been proposed that endometriosis is under the control of multiple genetic and environmental factors, much like the mechanisms underlying cancer [7]. Children and siblings (first-degree relatives) of women with endometriosis have a six-fold greater risk of developing endometriosis themselves [8]. In addition, genetic control of hormone levels, particularly low progesterone, could also influence the activity of disease [9]. Prolonged exposure to estrogen and obstruction of menstrual flow are other proposed environmental factors [10]. More recently, alteration of the vaginal biome in conjunction with retrograde menstruation has been proposed as another mechanism for endometriosis and seeding in the peritoneal cavity. An overload on the immune system that then fails to destroy endometrial cells outside the uterus is another proposed mechanism [11]. Surprisingly, up to 90% of women with endometriosis have been proposed to have an immunodeficiency [12,13]. This finding could help to explain why endometrial cells are not cleared from the peritoneal cavity and thorax.

One other interesting aspect of endometriosis is the frequent finding of elevated CA-125 levels with a moderate to severe disease burden. Our patient had a high level of CA-125 tumor marker in her blood. Although often associated with ovarian cancer, elevated CA-125 can also be found in many non-cancerous conditions [14] as well as other cancerous conditions such as lung cancer [15]. CA-125 is a surface glycoprotein, also known as Mucin-16, which is the largest membrane-associated mucin molecule that serves as a protective barrier for cells that possess the gene for it. As such, it can be found in the reproductive tract, lining cells in the respiratory tract, digestive tract, and cornea, among others. It has been promoted as a marker for endometriosis, although it is not very sensitive (52%) and not a good test for ruling out the disease. Almost half of women with endometriosis can be expected to have elevated levels of CA-125 (Mucin-16), and hence it might be useful as a marker for disease burden and recurrence. As might be expected, estrogen has been shown to promote CA-125 gene expression, and progesterone suppresses it [16]. This may be the basis, at least in part, for the growth of endometrial cells (and endometriosis) during the follicular phase of the ovarian cycle and its suppression during the luteal phase. Early investigation and treatment of pelvic endometriosis are essential for the prevention of thoracic spread [17]. Surgery, whether conservative or radical, and the administration of gonadotropin-releasing hormone analogues are essential for both catamenial [17] and abdominopelvic disease.

## Conclusions

Catamenial pneumothorax occurs in 3% to 6% of women with spontaneous pneumothorax, usually associated with pelvic endometriosis. Chest or scapular pain occurs in 90% of patients, and 33% have dyspnea. As far as the authors are aware, only one previous case report was reported in the literature with the patient being asymptomatic. It should be part of the differential diagnosis when a woman of reproductive age presents with spontaneous pneumothorax. Ascites secondary to endometriosis are rare, with approximately 60 cases being reported worldwide. Reported cases were mostly in African American (82%) women, making our case very peculiar as the patient was Asian and had both catamenial pneumothorax and abdominopelvic disease. Early investigation and treatment of pelvic endometriosis are essential for the prevention of thoracic spread. Surgery, whether conservative or radical and the administration of gonadotropin-releasing hormone analogues are essential for both catamenial and abdominopelvic disease. The choice of therapy depends on age, severity of disease, and desire for fertility.
